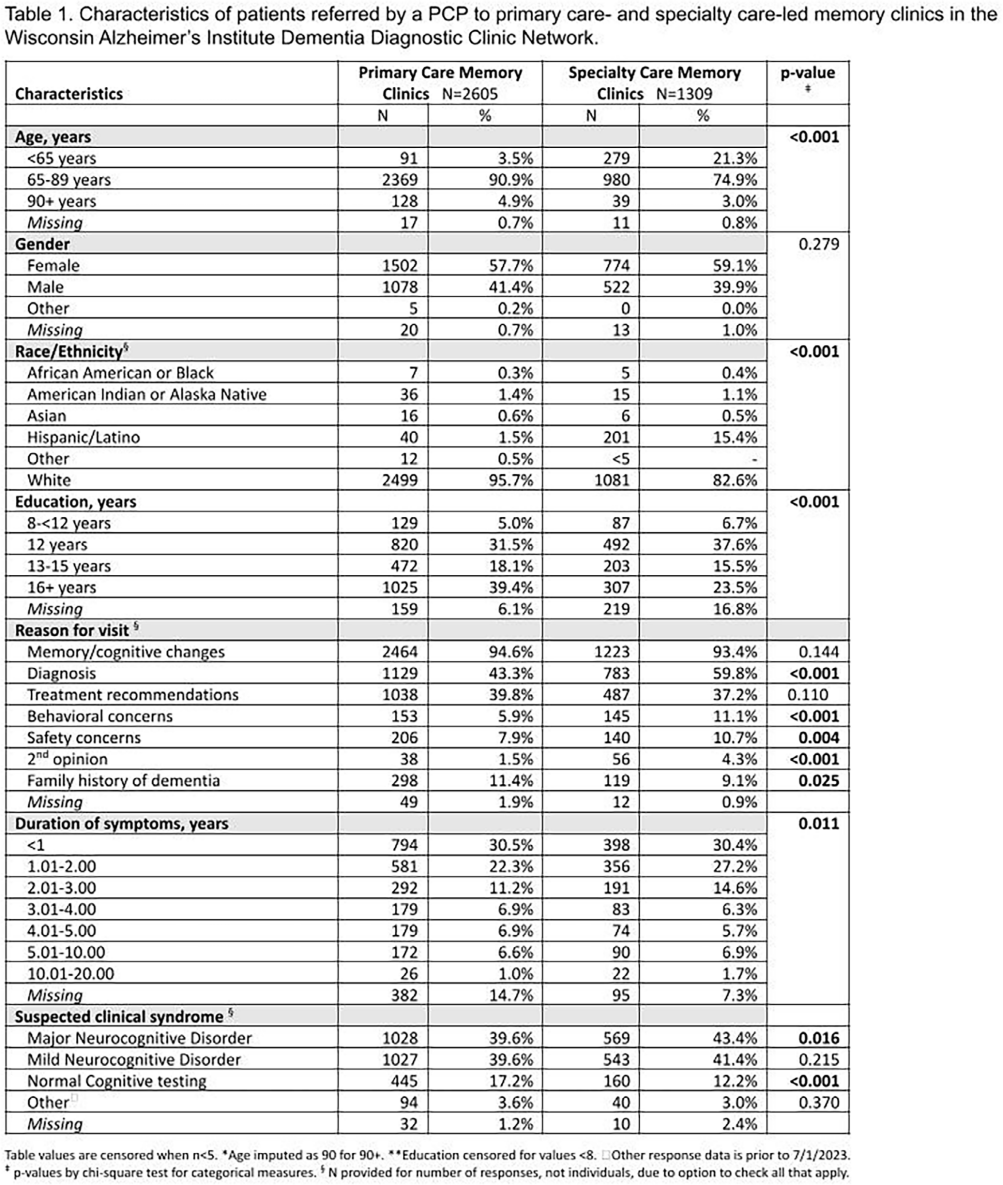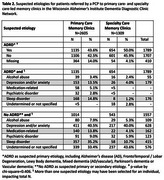# Differences in Patients Demographics and Etiologies in Primary vs. Specialty Care Memory Clinics: Insights from the Wisconsin Alzheimer's Institute Dementia Diagnostic Clinic Network

**DOI:** 10.1002/alz70860_107750

**Published:** 2025-12-23

**Authors:** Tamara J. LeCaire, Jennifer Landeta‐Vidal, Uriel Paniagua, Jody Krainer, Art Walaszek, Maria C Mora Pinzon, Cynthia M. Carlsson

**Affiliations:** ^1^ Wisconsin Alzheimer's Institute, University of Wisconsin School of Medicine and Public Health, Madison, WI, USA; ^2^ Wisconsin Alzheimer's Institute, University of Wisconsin School of Medicine and Public Health, Madison, WI, USA; ^3^ University of Wisconsin‐Madison School of Medicine and Public Health, Madison, WI, USA; ^4^ Wisconsin Alzheimer's Institute, Madison, WI, USA; ^5^ Wisconsin Alzheimer's Disease Research Center, University of Wisconsin School of Medicine and Public Health, Madison, WI, USA; ^6^ University of Wisconsin School of Medicine and Public Health, Madison, WI, USA; ^7^ Department of Medicine, Division of Geriatrics, School of Medicine and Public Health, University of Wisconsin‐Madison, Madison, WI, USA; ^8^ Wisconsin Alzheimer's Institute, University of Wisconsin ‐ Madison, Madison, WI, USA; ^9^ Division of Geriatrics and Gerontology, Department of Medicine, University of Wisconsin School of Medicine and Public Health, Madison, WI, USA

## Abstract

**Background:**

The Wisconsin Alzheimer's Institute Dementia Diagnostic Clinic Network (DDCN) serves as a community of practice for interdisciplinary memory clinics located in primary care‐ (internal medicine, family medicine, or geriatrics) or specialty‐clinics (neurology or psychiatry). We sought to examine differences in medical screenings performed, and suspected etiologies according to practice setting (primary care‐ vs specialty care‐based). Considering that most patients seen in DDCN clinics are referred by their PCPs, this analysis is restricted to those patients, to better understand what additional support primary care teams outside the network may need for making a diagnosis of ADRD.

**Methods:**

Cross‐sectional data submitted by DDCN clinics between 2021 and 2024 was analyzed. Data was collected during the patient's initial visit and included patient sociodemographics, screenings performed/needed, and suspected primary and secondary diagnoses. Data analyzed was only for patients who were referred to DDCN by a PCP. Descriptive analyses included chi‐square tests comparing diagnostic experiences by practice‐setting; and multivariate logistic regression modeling used to evaluate steps associated with making an ADRD diagnosis by practice will be presented.

**Results:**

17 primary care‐ and 11 specialty‐led clinics submitted data for 2,605 and 1,309 patients, respectively. Significantly different patterns emerged by setting (*p* = 0.001) for patient age, race/ethnicity, education level, reason for visit and suspected clinical syndrome (Table 1). Slightly more patients from DDCN primary care‐led clinics were subsequently suspected to have no cognitive impairment (17.2% vs 12.2%). Issues with sleep (e.g. sleep apnea) were identified more frequently in primary care‐based DDCN clinics (30.5% vs 20.5%, *p* <0.001). Among patients seen in primary‐care DDCN clinics, there was a higher proportion of suspected diagnosed with ADRD+depression (13.5% vs 4.0%), ADRD+sleep disorders (14.8% vs 1.2%), sleep disorders (35.2% vs 10.7%) or medication‐related cognitive decline (13.8% vs 4.1%) (Table 2).

**Conclusions:**

Findings suggest that DDCN primary care‐based clinics receive more patients with potentially reversible causes of cognitive decline, suggesting that changes in primary care diagnostic practices could reduce the need for referrals to specialty memory clinics. Increasing the capacity to diagnose and manage reversible causes of cognitive impairment within primary care could potentially lead to reduced wait times for specialty dementia care.